# *Akkermansia muciniphila* protects intestinal mucosa from damage caused by *S. pullorum* by initiating proliferation of intestinal epithelium

**DOI:** 10.1186/s13567-020-00755-3

**Published:** 2020-03-05

**Authors:** Linda Zhu, Xiaoxi Lu, Li Liu, Josef Voglmeir, Xiang Zhong, Qinghua Yu

**Affiliations:** 1 MOE Joint International Research Laboratory of Animal Health and Food Safety, College of Veterinary Medicine, Nanjing Agricultural University, Weigang 1, Nanjing, 210095 Jiangsu China; 2 Glycomics and Glycan Bioengineering Research Center (GGBRC), College of Food Science and Technology, Nanjing Agricultural University, Nanjing, China; 3 College of Animal Science and Technology, Nanjing Agricultural University, Weigang 1, Nanjing, 210095 Jiangsu China

## Abstract

*Akkermansia muciniphila*, a novel mucin-degrading bacterium, has been demonstrated to prevent the development of obesity and related complications. However, whether it can protect poultry from intestinal mucosal damage by enteropathogens has never been mentioned. In this study, we found that *A. muciniphila* colonized in the intestine and then relieved intestinal mucosal damage in chicks caused by *S. pullorum*, including anatomical and morphological damage, alleviation of body weight and intestinal inflammation. The repair process activated by *A. muciniphila* is accompanied by an increase in the number of goblet cells in the chick’s intestine and an up-regulation of Mucin 2 and trefoil factor 2 (Tff2). In addition, we also demonstrate that *A. muciniphila* improved colon length, crypt depth, increased the proliferating cell nuclear antigen, with the accelerated proliferation of intestinal epithelium through Wnt/β-catenin signaling pathway, thereby restoring the damaged intestinal mucosa. This study suggests that *A. muciniphila* activates the proliferation of intestinal cells protecting the intestinal barrier, thus relieving infection with *S. pullorum* in chickens.

## Introduction

*Salmonella* is an important zoonotic pathogen that not only infects livestock but also infects humans [1]. *Salmonella pullorum* (*S. pullorum*) can induce avian salmonellosis after infection in poultry, causing recessive infection and even causing death, which brings huge economic losses to the poultry industry. Antibiotics are always used to protect poultry from diseases like *S. pullorum* [2, 3], but recently antibiotic abuse has been a serious problem in China and even in the world [4, 5]. Antibiotics will not only bring potential hidden dangers to the safety of animal food, but also cause harm to human health and the living environment [6, 7]. Nowadays, people are trying to use probiotics as a replacement of antibiotics and many conventional probiotics such as *Lactobacillus* and *Bacillus subtilis* have emerged in the market [8]. However, due to the limitation of currently used probiotics, new promising probiotics are still worth exploring in the future.

*Akkermansia muciniphila* (*A. muciniphila*) is a gram-negative, non-motile, non-spore-forming, oval-shaped bacterium, which is able to use mucin as its sole source of carbon and nitrogen [9]. *A. muciniphila* is culturable under anaerobic conditions on medium containing gastric mucin, and is able to colonize the gastrointestinal tracts of a number of animal species [10]. It is known as a next generation beneficial microbe for which it has been proven that it can prevent the development of obesity and associated complications [9]. Although *A. muciniphila* is in close contact with the intestinal epithelial, it has never been reported before whether it can protect poultry from intestinal mucosa damage.

The intestinal mucosal barrier is composed of a mechanical barrier, a chemical barrier, an immune barrier and a biological barrier, which can prevent harmful substances such as bacteria and toxins from entering the body through the intestinal mucosa [11, 12]. Once the intestinal mucosal barrier is impaired causing increased intestinal permeability and intestinal microbiota imbalance, it can lead to bacterial and endotoxin translocation [13], and can induce and aggravate systemic inflammatory response and multiple organ dysfunction [14, 15].

Several studies have shown that intestinal stem cells (ISC), which are located at the base of the intestinal crypts, play important roles in protecting the intestinal mucosa barrier by governing proliferation and differentiation of the intestinal epithelium [13, 16]. In addition, the integrity of mucosal epithelial function has been demonstrated to play a key role in resisting pathogens. A previous study demonstrated that *Lactobacillus* could accelerate ISC regeneration to protect the integrity of intestinal mucosa [17] and induce ISC differentiation into goblet cells [18]. Here, we show that *A. muciniphila* has a critical role in directing the intestinal stem cells to improve the intestinal mucosa damage by *S. pullorum* by modulation of the Wnt/β-catenin signaling pathway that regulates the proliferation of the intestinal epithelium.

## Materials and methods

### Animals and bacteria strains

Chicken studies were approved by the Institutional Animal Care and Use Committee of Nanjing Agricultural University. The *A. muciniphila* ATCC BAA-835 strain was presented by Li Liu from Food science and technology of Nanjing Agricultural University.

**Figure 1:**
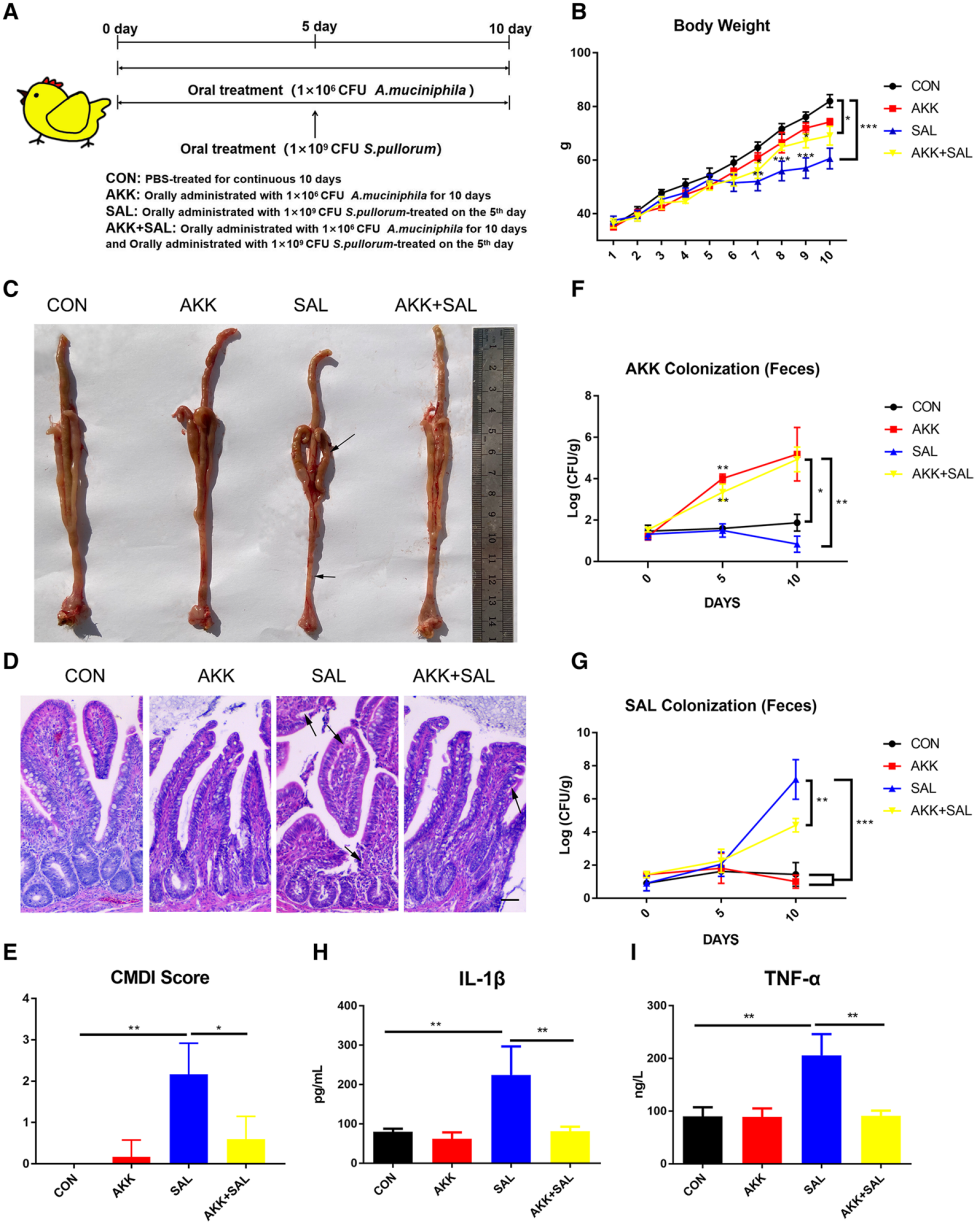
***A. muciniphila*ameliorates*S. pullorum*-induced intestinal mucosa damage in chicks. A** Newborn chicks were orally administrated with 200 μL PBS or *A. muciniphila* (10^6^ CFU) suspended in 200 μL PBS once a day, for a period of 10 days. Chicks were orally administered *S. pullorum* (10^9^ CFU) on the fifth day. On the 10^th^ day, chickens were sacrificed and subsequent experiments were performed. **B** Changes in chicks’ body weight were monitored each day; *n* = 12. **C** The anatomical morphology of chicks treated with PBS, *A. muciniphila*, *S. pullorum* or *A. muciniphila* plus *S. pullorum*. The areas marked with an arrow show where the colon became shorter, thinner and transparent, and the cecum abscess. **D** Photomicrographs of the chick colons. The areas marked by the arrow were villous shedding and colonic epithelial cell damage caused by *S. pullorum*. Scale bars 200 μm. **E** CMDI score of different groups. **F** and **G** Chicks were orally administered *A. muciniphila* (10^6^ CFU), a standard curve of *A. muciniphila* was established and chicks’ stool DNA was extracted from fresh manure, the number of *A. muciniphila* in chick feces at the indicated time points was detected by quantitative RT-PCR. **H** The expression of IL-1β in the four groups was detected using an ELISA kit; *n* = 12. **I** The expression of TNF-α in the four groups was detected using an ELISA kit; *n* = 12.

Newborn chicks were orally administered *A. muciniphila* (10^6^ CFU) suspended in 200 μL PBS once a day, for a period of 10 days. Chicks were orally administered *S. pullorum* on the 5^th^ day. The detailed animal treatment methods are listed in Figure 1A. The body weights of the chicks were recorded. On the 10^th^ day, the chicks were sacrificed, their colons were removed and the colon length was measured. The crypt depth of the colon was measured by image J software. Histological pathology was detected under light microscopy.

### Cytokine detection

Colons were collected from euthanized chicks, and organs were then homogenized and spun down. The supernatant was stored at −20 °C until use for cytokine analysis. Interleukin 1β (IL-1β) and tumor necrosis factor alpha (TNF-α) were measured using an ELISA kit according to the manufacturer’s instructions.

### Quantitative RT-PCR

The colon samples of different treatment groups were trimmed to a length of 1 cm, and total RNA was extracted from colon samples using RNAiso Plus (Takara, Dalian, China). Reverse transcription of the RNA was performed with the primers listed in Table 1. Two microliters of template RNA were reacted with TagMan PCR Master Mix for a final volume of 20 µL (Takara). The thermal cycling conditions were 5 min at 95 °C, followed by 40 cycles of 15 s at 95 °C and 34 s at 60 °C using an Applied Biosystems 7500 real-time PCR system.

**Table 1:** **The primers used in this study**

Primer names	Forward	Reserve
Chicken-GAPDH	GGCACGCCATCACTATC	CCTGCATCTGCCCATTT
Chicken-Muc2	ATTGTGGTAACACCAACATTCATC	CTTTATAATGTCAGCACCAACTTCTC
Chicken-Tff2	CTGAACAGCAATAACCACCC	TAATCCCCACAGAGACCACA
Chicken-Wnt3	GAAGCTGCGAGGTCAA GACT	TTGCACGTTCTGTCCCTTGT
Chicken-Lgr5	TACGTCTTGCAGGAAATGGCT	GGAACCTGGCGTAGTTGGTTA
Chicken-Axin2	GGGCTGGGGAGCTTAAAAGT	TCACTATCGTTTGCGCTGGT
Chicken-Lrp5	GCAAGAGCGAGCTCCCAAGA	AGGCCCATTGGCTGAAGGAT
Chicken-GSK3ß	TCCATTCCTTTGGGATCTGCC	TACACAGCCCGCTGACCACA
Chicken-TCF3	GGAATGCTGATGTGGGACCG	CCCAAACTGTGGGACCGAAA

### Immunofluorescence assay

A 1-cm section of colon was collected from different groups, fixed overnight in 4% paraformaldehyde, embedded in paraffin wax and sectioned at 5 μm. The colon sections were then permeabilized with 0.5% Triton X-100 for 15 min, followed by washing three times with HBSS and incubation for 1 h in 3% BSA in HBSS to reduce nonspecific background. For β-catenin and proliferating cell nuclear antigen (PCNA) staining, colon sections were permeabilized and incubated with β-catenin antibody (Ser45.D2U8Y) (1:200, Cell signaling technology, Danvers, USA) and anti-mouse PCNA antibody (1:200, Abcam, Shanghai, China) respectively overnight and then incubated again in dylight-488-conjugated goat anti-rabbit IgG and goat anti-mouse IgG (H + L) PE conjugated respectively as a secondary antibody. The slides were then stained with DAPI (1:1000, Invitrogen, Shanghai, China) for 10 min at room temperature. The samples were examined with a Zeiss 710 laser scanning confocal microscope. Fluorescence images were collected for further qualitative and quantitative analysis. The mean fluorescence intensity of β-catenin and PCNA was analyzed by image J software.

### Periodic acid Schiff (PAS) staining

A 1-cm section of colon was collected from each chick of different groups, fixed in 4% paraformaldehyde overnight, embedded in paraffin and sectioned in 5 μm and dewed in xylene for 7 min, repeated 2 times, then placed at 100%, 90% per step for 2 min, 75% ethanol and water. It was then treated with periodic acid for 5–10 min and rinsed with running water for 5 min to dry excess water on the sections. The Schiff dye solution and the dye were added for 10–15 min, and then the water was rinsed for 5 min and subjected to conventional dehydration, transparency, and sealed.

### Statistical analysis

Results are expressed as mean ± SD. One-way ANOVA was employed to determine statistical differences among multiple groups, *P < 0.05, **P < 0.01, and LSD post-tests were used to determine inter-group differences.

## Results

### *A. muciniphila* ameliorates *S. pullorum*-induced intestinal mucosa damage in chicks

To investigate the possible role of *A. muciniphila* in chicks, chicks were orally administered with PBS (200 μL) as the control or *A. muciniphila* (10^6^ CFU) suspended in 200 μL PBS once a day for 10 days and orally treated with *S. pullorum* (10^9^ CFU) suspended in 200 μL PBS on the fifth day (Figure 1A). *S. pullorum* infection significantly reduced body weight compared with the control group, while *A. muciniphila* partly improved the body weight loss (Figure 1B). *S. pullorum* also caused a severe mucosal damage with shorter colon, thinner and more transparent cecum (Figure 1C). Histological examination also shows a higher level of villous shedding and colonic epithelial cell damage when treated with *S. pullorum* alone (Figure 1D). CMDI (Colon mucosa damage index) score is an evaluation index for gross damage of specimens [19], showing that *S. pullorum* treatment had a severe colon mucosal damage and pretreating with *A. muciniphila* could resist this kind of damage (Figure 1E).

Moreover, the amounts of *A. muciniphila* and *S. pullorum* in feces were detected according to the standard curves of bacteria. We found *A. muciniphila* reached 10^5^ CFU/g in feces at the 10^th^ day (Figure 1F), while it reduced *S. pullorum* colonization (Figure 1G). The protein levels of pro-inflammatory cytokines IL-1β and TNF-α were determined as markers for inflammation (Figures 1H, I). Consistent with body weight results, the chicks’ IL-1β and TNF-α were kept at lower levels compared with those treated with *S. pullorum* when treated with *A. muciniphila*, suggesting that *A. muciniphila* can significantly reduce inflammation level. Together, these data indicate that chicks treated with *S. pullorum* probably lead to severe intestinal mucosa damage while oral administration of *A. muciniphila* can accelerate chicks’ body growth and relieve this intestinal mucosa damage.

### *A. muciniphila* stimulates goblet cells and the mRNA expression of mucin proteins

**Figure 2:**
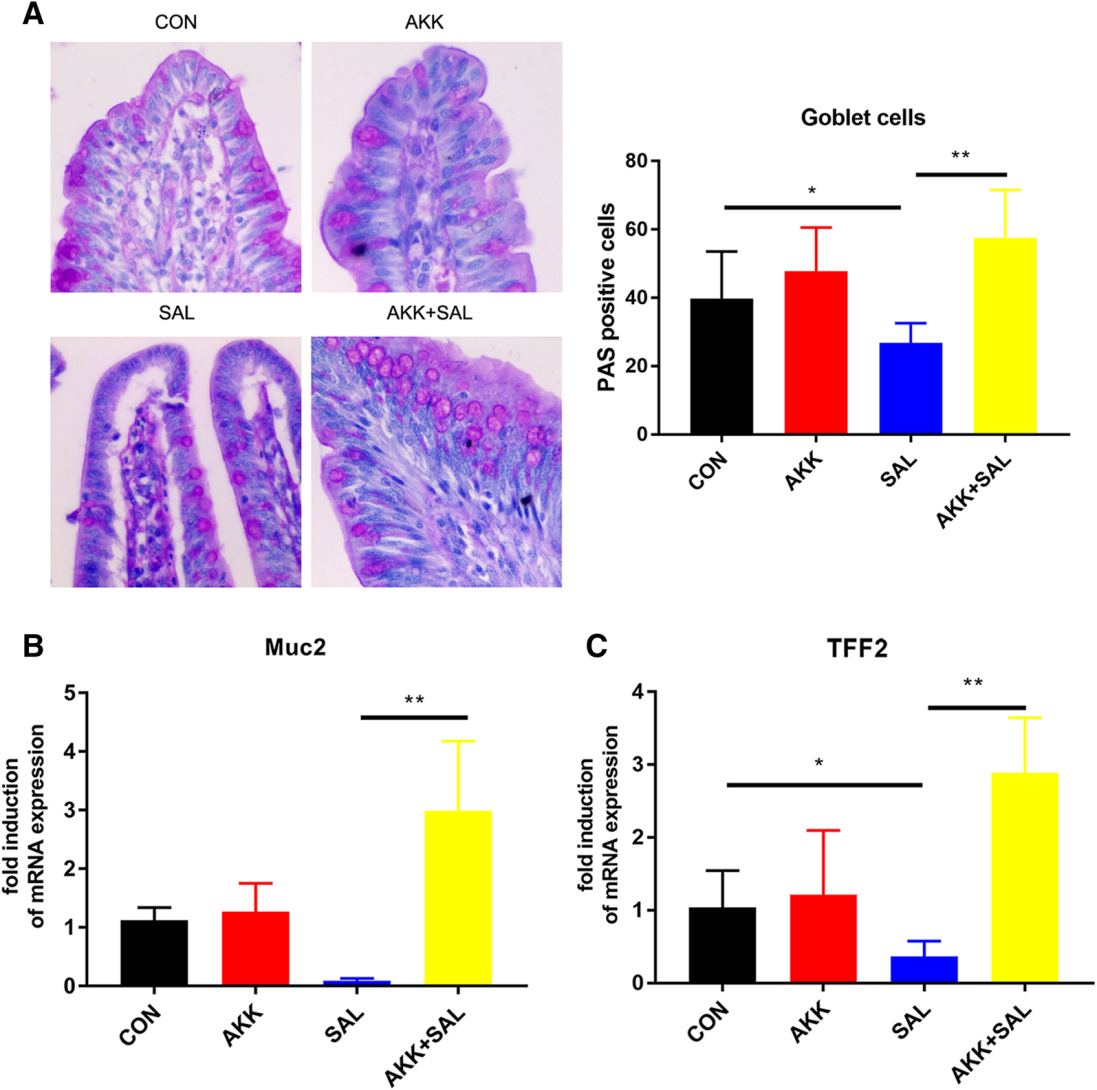
***A. muciniphila*secretes mucin protein and up-regulates the expression of related genes. A** Photomicrographs of the colons of chicks treated with PBS, *A. muciniphila*, *S. pullorum* or *A. muciniphila* plus *S. pullorum*. The number of goblet cells in each crypt was detected. More than twenty crypts were recorded. Scale bars 50 μm. **B**, **C** The fold induction of relative mRNA expressions of Muc2 and Tff2 in chicks’ colon; *n* = 12.

Since *A. muciniphila* can relieve this intestinal mucosa damage caused by *S. pullorum*, we subsequently studied how *A. muciniphila* works. Goblet cells are mucus secreting cells scattered in the intestinal mucosa. Their main function is to secrete mucin, which plays an important role in the intestinal barrier. Since *A. muciniphila* is a typical intestinal mucin-degrading bacterium, we used PAS staining to detect intestinal goblet cells. Chicks treated with *S. pullorum* had a great loss of goblet cells on the intestinal surface, while *A. muciniphila* reversed this situation and significantly increased goblet cell distribution (Figure 2A). Mucin 2 (Muc2) is a secreted mucin that is expressed in the colon, small intestine and airway epithelial cells. The protein forms a layer of mucus on the surface of the intestine to lubricate and antagonize the intestinal adhesion and invasion of pathogenic bacteria [20, 21]. Trefoil factor 2 (Tff2) are stable secretory proteins expressed in the gastrointestinal mucosa. Their functions are not defined, but they may protect the mucosa from insults, stabilize the mucus layer and affect healing of the epithelium [22, 23]. We examined these two gene expressions in chicks’ colon and found a significant up-regulation after treatment with *A. muciniphila* (Figures 2B, C). In general, *S. pullorum* induced the loss of a large amount of secreted mucin, but *A. muciniphila* secreted mucin and up-regulated the expression of related genes, thereby repairing intestinal mucosal damage.

### *A. muciniphila* activates gut growth and increases the proliferation of intestinal epithelium after damage

**Figure 3:**
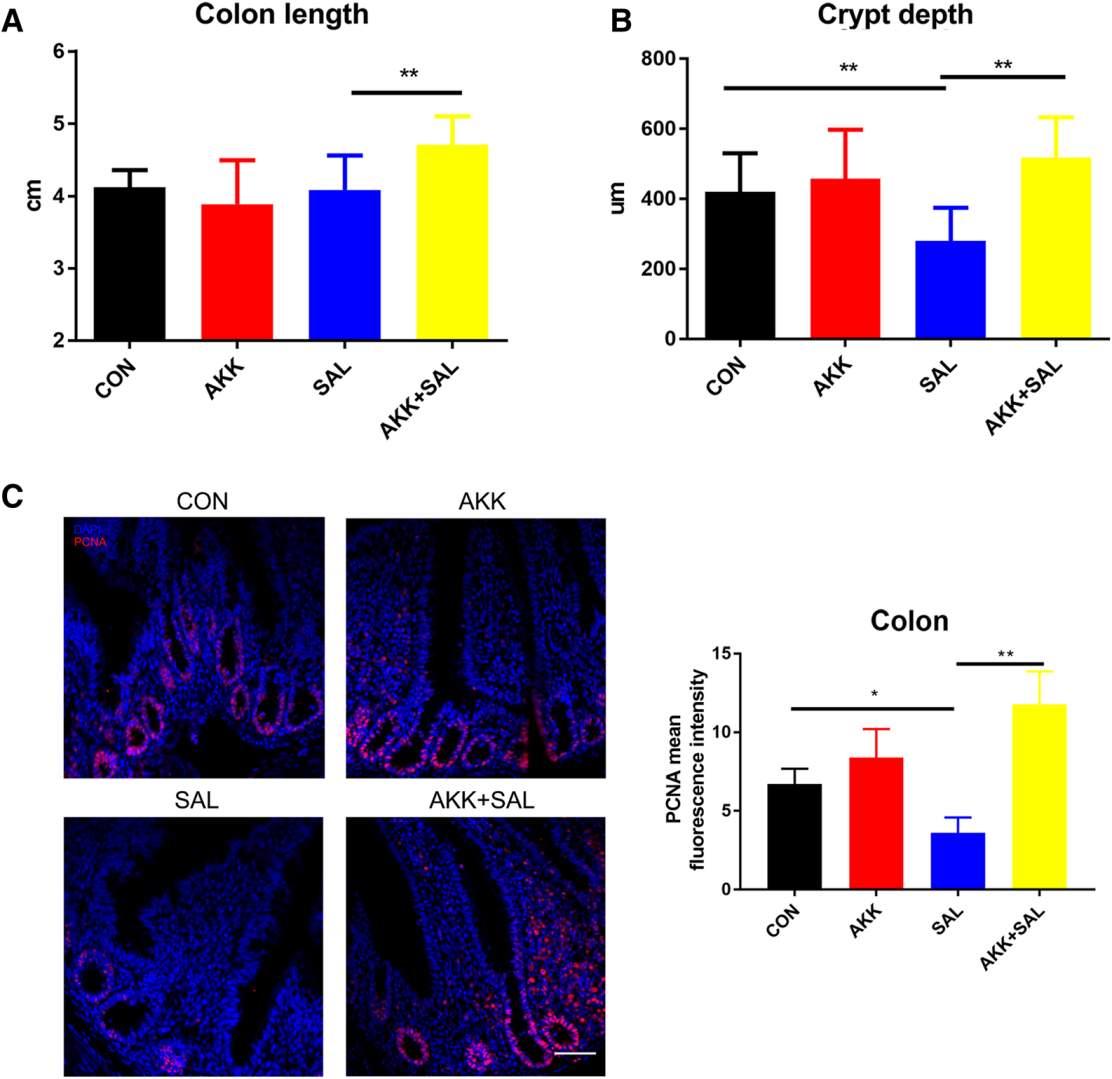
***A. muciniphila*activates gut growth and increases the proliferation of intestinal ISC after damage. A** The colon lengths of chicks from the four treated groups. Treatment with *A. muciniphila* after *S. pullorum* significantly increased the colon length compared to *S. pullorum*. **B** Segments of the colon were processed to measure the crypt depth. Administration of *S. pullorum* decreased the crypt depth compared to the control group and treated with *A. muciniphila* significantly increased crypt depth. **C** Confocal images (PCNA staining, red; and DAPI staining, blue) of colon in intestine, more than 20 crypts were recorded, and the fluorescence intensity of PCNA was detected. Scale bars 200 μm.

Compared with the control group, chicks treated with *S. pullorum* had reduced colon length, while *A. muciniphila* significantly increased colon length (Figure 3A), indicating that *A. muciniphila* can increase intestinal proliferation. Histological examination shows that the crypt depth of chicks treated with *S. pullorum* was much shallower than that of the untreated group, while those treated with *A. muciniphila* returned to a normal level (Figure 3B). We then naturally associated these phenomena with the proliferation of ISC. Immunofluorescence assays show that chicks pretreated with *A. muciniphila* before *S. pullorum* infection had higher PCNA fluorescence intensity in the crypt, further validating our conjecture (Figure 3C).

### *A. muciniphila* regulates the proliferation of intestinal stem cells through the Wnt/β-catenin signaling pathway

**Figure 4:**
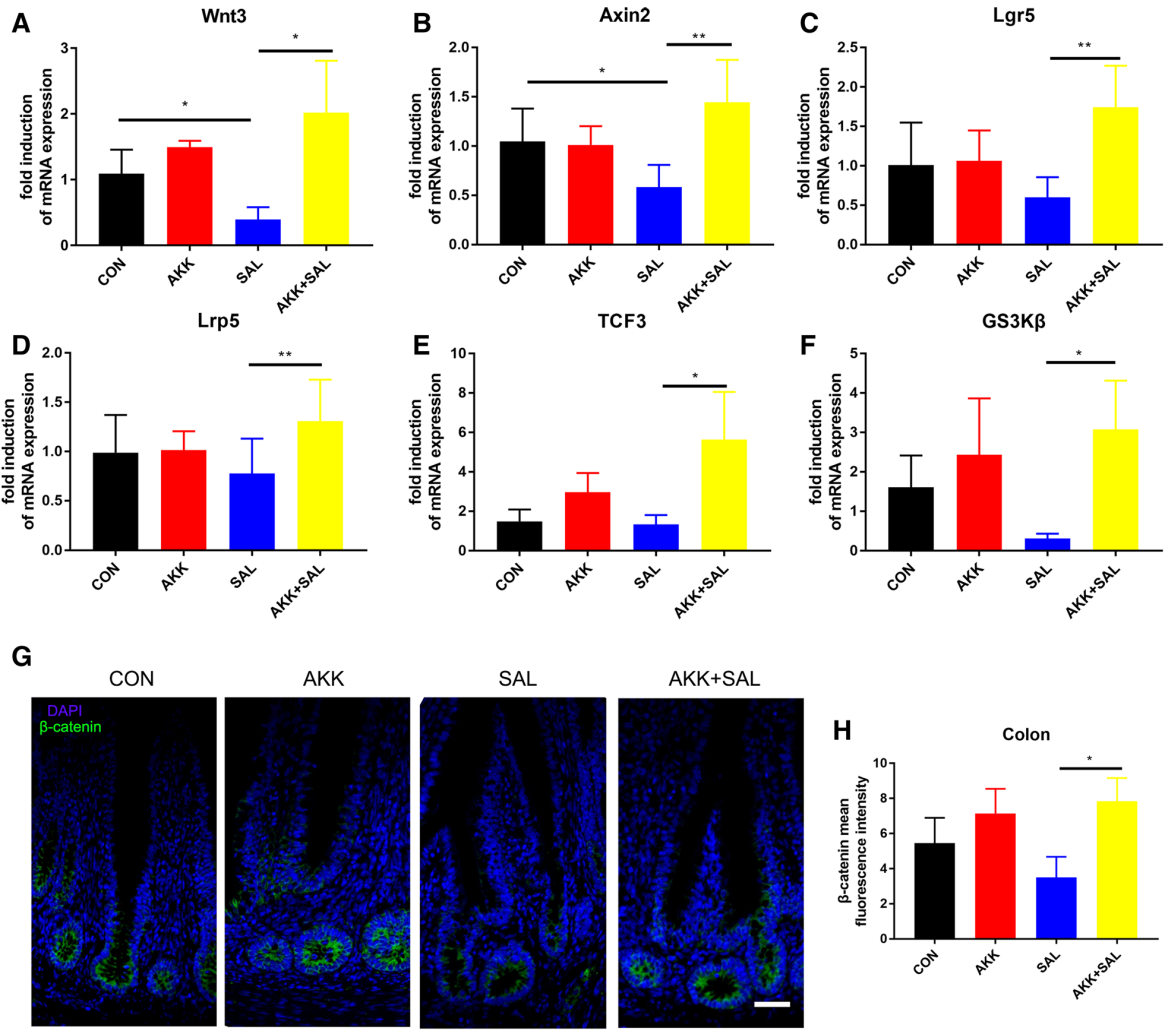
***A. muciniphila*regulates the proliferation of intestinal stem cells though Wnt/β-catenin signaling pathway**. **A**–**F** The fold induction of relative mRNA expressions of Wnt3, Axin2, Lgr5, Lrp5, TCF3 and GS3Kβ in chicks’ colon; *n* = 12. **G**, **H** Confocal images (β-catenin staining, green; and DAPI staining, blue) of colon, more than 20 crypts were recorded. The β-catenin fluorescence intensity was detected. Scale bars 200 μm.

The Wnt/β-catenin signaling pathway was reported previously as a key factor in maintaining crypt cell proliferation [24] and ISC were critical for damage-induced intestinal regeneration [25]. We detected the up-regulation of PCNA at intestinal stem cells in chicks treated with *A. muciniphila* after *S. pullorum* induced damage. To further explore the underlying mechanisms of how *A. muciniphila* regulates the proliferation of intestinal stem cells, we wanted to detect whether the Wnt/β-catenin signaling pathway was activated. As shown by the results from real-time quantitative PCR, there was a significant up-regulation on the mRNA expression levels of ISCmarkers and downstream to related genes of Wnt, axin-like protein 2 (Axin2), leucine-rich repeat-containing G-protein coupled receptor 5 (Lgr5), low-density lipoprotein receptor-related protein 5 (Lrp5), transcription factor 3 (TCF3) and glycogen synthase kinase-3β (GS3Kβ) (Figures 4A-F). Next, we also found A*. muciniphila* induced a higher β-catenin fluorescence intensity compared to the chicks with *S. pullorum* treated alone (Figures 4G, H). All these data indicate that *A. muciniphila* could alleviate intestinal mucosal damage caused by *S. pullorum* by regulating the proliferation of IEC through the Wnt/β-catenin signaling pathway.

## Discussion

Due to its beneficial effects on obesity and type-2-diabetes, a *Verrucomicrobia* bacterium, *A. muciniphila*, is considered to be a next-generation probiotic [13, 26]. There were also reports showing that colonization by *A. muciniphila* is associated with intestinal integrity [9], but it is still a controversy and has never been reported before whether it can protect poultry from intestinal mucosal damage. The results of this study indicate that *A. muciniphila* plays a crucial role in the proliferation of chick intestinal stem cells while relieving the intestinal mucosa damage caused by *S. pullorum* through the Wnt/β-catenin signaling pathway.

There are billions of bacteria in the intestines, and normally they stay balanced within the intestine [27]. When the intestinal mucosa is damaged, pathogens are more likely to invade the body [28]. *A. muciniphila* is one of the most abundant members of the human gut microbiota [29]. It resides in the mucus layer of the large intestine, where it is involved in maintaining intestinal integrity [9]. It differs from other common probiotics; *A. muciniphila* is a Gram-negative bacterium, but its colonization is believed to have anti-inflammatory effects in humans as in chicks [26]. It has been reported that induced acute enteritis in chicks will change the composition of intestinal microbiota [30]. There is also a report indicating that improving the proportion of beneficial bacteria in the intestinal tract of infected chickens [31, 32] can reduce the levels of pro-inflammatory cytokines IL-1β and TNF-α, and reduce intestinal inflammation [33]. In this study, we first demonstrate that *A. muciniphila* could reduce inflammation levels caused by *S. pullorum* infection in chicks and relieve intestinal mucosal damage.

There are still many controversies about the effects of *A. muciniphila* in different animals and diseases. It has been previously reported that *A. muciniphila* exacerbates gut inflammation in *S. typhimurium*-infected gnotobiotic mice [34]. It is noteworthy that *S. typhimurium* is a lethal pathogen to mice, and *S. pullorum* infection only causes diarrhea in chickens without death. Under serious intestinal damage caused by *S. typhimurium*, *A. muciniphila* as a gram-negative bacteria with lipopolysaccharides (LPS) on their inner wall may exacerbate intestinal injury. This is totally different from *S. pullorum* infection in chickens. In fact, the host animals (mice and chickens) are also different, which may also explain the different results of *A. muciniphila.* Finally, since *A. muciniphila* colonized the colon, most of the data that we tested in this experiment came from the colon, but all of the data in that article came from the cecum; therefore, combining the above results may explain this difference.

Goblet cells are simple columnar epithelial cells that secrete gel-forming mucins, like mucin Muc2 in the intestine, which secretes mucus in order to protect the intestinal mucosa barrier [33]. The importance of the mucus layer was emphasized in studies using Muc2 knockout mice, which did not have a colonic mucus layer covering the intestinal epithelial layer [35]. These mice suffered from a decreased intestinal barrier function, an increased inflammatory status and had signs of colitis [35]. *Salmonella* invades the top of the villi, enters and propagates in the epithelial cells, infects adjacent cells or enters the lamina propria, destroying the intestinal mucosal barrier. We found that *S. pullorum* inhibited the production of goblet cells and decreased the expression of Muc2 and Tff2, which caused serious intestinal mucosa damage. It has been previously reported that *A. muciniphila* can increase mucus thickness and this ability is associated with the secretory function of goblet cells [36]. In our study, treatment with *A. muciniphila* after damage restored the number of goblet cells, reversing intestinal mucosa damage and increasing the expression of Muc2 and Tff2, which was also confirmed in a previous report [31].

It is well-known that the Wnt/β-catenin signaling pathway is a central regulator of development and tissue homeostasis [37]. And in the intestine, Wnt signaling is also known as a principal organizer of epithelial stem cell identity and proliferation [38]. It was also reported that crypt base columnar (CBC) cells that characteristically express the R-spondin receptor Lgr5 were identified as the main stem cell population in the gut and had the ability to produce all lineages of Intestinal epithelial cells [38]. Crypts are the driving force behind the perpetual renewal of the intestinal mucosa barrier, and the Wnt/β-catenin signaling pathway is necessary for stem cells in the crypts to survive [39, 40]. Hence, based on our findings, we now add evidence that *A. muciniphila* might protect against *S. pullorum* induced intestinal mucosa damage by initiating the proliferation of intestinal stem cells though the Wnt/β-catenin signaling pathway.

In summary, we found that *A. muciniphila* could relieve intestinal mucosal damage caused by *S. pullorum* by secreting mucin protein and increasing the proliferation of intestinal stem cells though the Wnt/β-catenin signaling pathway.
